# High-Entropy Alloy-Engineered ZnIn_2_S_4_ Photocatalyst for Efficient In Vitro Eradication of MRSA and Inhibition of Biofilm Formation via ROS-Mediated Mechanisms

**DOI:** 10.3390/ijms27209073

**Published:** 2026-10-13

**Authors:** Xuehua Chen, Tai-Cheng Zhou, Changle Wen, Ying Zhang, Hao Wang, Jia Wei

**Affiliations:** 1 School of Medicine, Yunnan University, Kunming 650091, China; chenxh1117_edu@163.com (X.C.); wenchangle2000@163.com (C.W.); 2 Central Laboratory, The Affiliated Hospital of Yunnan University, Kunming 650021, China; zhoutc@ynshhyy.com; 3 Key Laboratory of Bioinorganic and Synthetic Chemistry of the Ministry of Education, Fine Chemical Industry Research Institute, School of Chemistry, Sun Yat-Sen University, Guangzhou 510275, China; zhangy656@mail2.sysu.edu.cn; 4 Faculty of Chemical Engineering, Kunming University of Science and Technology, Kunming 650500, China

**Keywords:** high-entropy alloys (HEAs), ZnIn_2_S_4_, photocatalytic antibacterial, antibiofilm, MRSA

## Abstract

Antibiotic-resistant bacterial infections, particularly those caused by methicillin-resistant *Staphylococcus aureus* (MRSA), pose a critical clinical challenge and necessitate alternative non-antibiotic antimicrobial strategies. Herein, a high-entropy alloy-modified ZnIn_2_S_4_ (HEA–ZIS) photocatalytic nanoplatform was rationally engineered and evaluated for in vitro antibacterial performance. FeCoNiCuZn high-entropy alloy nanoparticles were uniformly anchored onto hierarchical ZnIn_2_S_4_ nanoflowers via a combined solvothermal–impregnation strategy. Structural and physicochemical characterizations reveal that the intrinsic flower-like morphology is well retained, while HEA incorporation induces lattice distortion, multivalent metal states, and interfacial charge redistribution. The optimized 3%HEA–ZIS exhibits potent antibacterial activity against MRSA under visible light, achieving 98.30% inactivation and a 5.91-log reduction, with a low minimum bactericidal concentration (0.30 mg mL^−1^). It also demonstrates strong antibiofilm activity by inhibiting MRSA biofilm formation, reducing biofilm biomass by over 84%, while maintaining good in vitro biosafety. Mechanistic studies reveal enhanced light absorption, improved charge separation, and accelerated interfacial electron transfer, promoting efficient oxygen activation. Electron paramagnetic resonance and in situ DRIFTS analyses identify a cascade reactive oxygen species (ROS) pathway responsible for the enhanced antibacterial efficacy. Overall, this work provides insights into high-entropy alloy-engineered semiconductor nanoplatforms and demonstrates their potential as promising biomedical nanomaterials for non-antibiotic antimicrobial applications.

## 1. Introduction

Antibiotic-resistant bacterial infections have become a critical global health challenge, leading to increased morbidity, mortality, and substantial healthcare burden [1,2,3]. Among these pathogens, methicillin-resistant *Staphylococcus aureus* (MRSA) is particularly problematic due to its ability to cause persistent and hard-to-treat infections, including chronic wounds, implant-associated infections, and bloodstream infections [4,5,6]. The limited efficacy of conventional antibiotics against MRSA is mainly due to its diverse resistance mechanisms, including target modification, reduced membrane permeability, and active efflux of antimicrobial agents [7,8,9]. These mechanisms significantly diminish antibiotic susceptibility and compromise therapeutic outcomes. This challenge is further exacerbated by the formation of bacterial biofilms, which provide an additional layer of protection [10,11]. Within biofilms, bacteria are encapsulated in a self-produced extracellular polymeric substance (EPS) matrix that acts as a protective barrier, significantly restricting antibiotic penetration and shielding bacteria from host immune responses [12,13]. This highly organized microenvironment markedly enhances bacterial tolerance and facilitates horizontal gene transfer, thereby accelerating the propagation of antibiotic resistance [14,15]. As a result, biofilm-associated MRSA infections are notoriously difficult to eradicate and are frequently linked to chronic and recurrent disease states [16,17]. Therefore, there is an urgent need to develop efficient, biocompatible, and non-antibiotic antimicrobial strategies that can effectively eliminate antibiotic-resistant bacteria while simultaneously suppressing biofilm formation.

Photocatalysis-based antimicrobial therapy has emerged as a promising non-antibiotic alternative to conventional antibacterial approaches [18,19,20]. This strategy utilizes semiconductor materials to harvest light energy and generate electron–hole pairs, which subsequently drive interfacial redox reactions to produce reactive oxygen species (ROS), such as superoxide radicals (•O_2_^−^), hydroxyl radicals (•OH), and singlet oxygen (^1^O_2_) [21,22,23,24]. These ROS can non-selectively oxidize cellular components, including membranes, proteins, and nucleic acids, leading to rapid microbial inactivation [25,26,27]. Compared with conventional antibiotics, ROS-mediated antimicrobial strategies offer compelling advantages, including broad-spectrum activity, reduced likelihood of resistance development, and spatiotemporal controllability under mild conditions [28,29,30,31]. Among various photocatalysts, zinc indium sulfide (ZnIn_2_S_4_, ZIS) has attracted considerable attention due to its unique physicochemical properties [32,33,34,35]. ZIS is a ternary chalcogenide semiconductor with a moderate bandgap (~2.06–2.85 eV), enabling efficient absorption of visible light. Its electronic band structure is well-suited for photocatalytic reactions, providing sufficient thermodynamic driving force for oxygen reduction and ROS generation [36,37,38,39]. Additionally, ZIS exhibits favorable biocompatibility, low toxicity, and good chemical stability, making it a promising candidate for biomedical antimicrobial applications [40,41,42]. However, despite these advantages, pristine ZIS suffers from several intrinsic drawbacks, including rapid recombination of photogenerated electron-hole pairs, limited density of surface-active sites, and suboptimal charge transfer efficiency [43,44]. These intrinsic limitations restrict ROS generation, thereby limiting the antibacterial performance of pristine ZIS, especially against antibiotic-resistant strains and biofilm-associated bacteria.

To overcome these challenges, various modification strategies have been explored, such as heterojunction engineering, defect introduction, and elemental doping [45,46,47]. Recently, high-entropy alloys (HEAs) have emerged as a novel class of catalytic materials with exceptional physicochemical properties. HEAs are typically composed of five or more principal elements in near-equal atomic ratios, forming solid-solution alloys stabilized by high configurational entropy [48,49]. This unique compositional complexity gives rise to remarkable features, including severe lattice distortion, abundant defect sites, and sluggish diffusion, which collectively enable tunable electronic structures, enhanced catalytic activity, and rich active sites [50,51,52,53]. In the context of photocatalytic antibacterial applications, HEAs offer several distinct advantages. The multimetallic composition facilitates efficient electron redistribution and interfacial charge transfer, thereby suppressing charge recombination. The intrinsic lattice distortion and defect-rich nature of HEAs can serve as effective charge trapping and activation centers, prolonging carrier lifetimes and promoting ROS generation. Moreover, the diverse elemental composition provides multiple catalytic sites for oxygen activation, enhancing the formation of reactive species [54,55,56,57]. Among various HEA systems, equiatomic FeCoNiCuZn is particularly attractive owing to the complementary functionalities of its constituent elements [58]. Fe and Co can provide rich redox couples (e.g., Fe^2+^/Fe^3+^ and Co^2+^/Co^3+^) that facilitate Fenton-like reactions and promote the generation of hydroxyl radicals, while Ni offers abundant active sites favorable for charge transfer [59,60]. Cu not only enhances the overall electrical conductivity but also imparts intrinsic antibacterial activity through ion release and contact-mediated effects [61]. Meanwhile, the presence of Zn is expected to improve lattice compatibility with the ZnIn_2_S_4_ matrix, thereby reducing interfacial strain and enabling the formation of an intimate heterojunction [62]. More importantly, the coexistence of multiple 3d transition-metal elements in FeCoNiCuZn HEA can induce significant d-orbital hybridization and electronic coupling, leading to continuous modulation of the local electronic environment and redistribution of electron density [63,64]. Such multielement orbital interactions are beneficial for optimizing adsorption/desorption behavior of oxygen-containing intermediates, lowering interfacial reaction energy barriers, and promoting directional charge transfer. Motivated by these considerations, the incorporation of FeCoNiCuZn HEA nanoparticles into ZnIn_2_S_4_ matrices represents a promising pathway to construct highly efficient photocatalytic antibacterial systems. Such hybrid structures are expected to exhibit synergistic effects, including improved visible-light absorption, optimized band structure, enhanced charge separation and transfer, and increased surface reactivity [65,66]. Nevertheless, despite the growing interest in both ZIS-based materials and FeCoNiCuZn HEA systems, their integration for antimicrobial applications remains largely unexplored. In particular, a systematic understanding of how HEA incorporation influences optical properties, electronic structure, ROS generation pathways, and antibacterial performance is still lacking.

In this work, FeCoNiCuZn high-entropy alloy-modified ZnIn_2_S_4_ composites (HEA–ZIS) were rationally designed and synthesized via a two-step solvothermal and impregnation assembly strategy. The structural, morphological, optical, and surface chemical properties of the composites were systematically characterized. The photocatalytic antibacterial and antibiofilm performances were evaluated using methicillin-resistant *Staphylococcus aureus* (MRSA) cells. Particular emphasis was placed on elucidating the underlying mechanisms, including band structure evolution, charge carrier dynamics, oxygen activation behavior, and ROS generation pathways, supported by techniques such as Mott–Schottky analysis, electrochemical measurements, in situ DRIFTS, and EPR spectroscopy. In addition, the in vitro biosafety of the composites was systematically assessed. By integrating materials science with microbiology, this study establishes a structure–property–mechanism framework that guides the rational design of high-entropy alloy-engineered semiconductor nanoplatforms and suggests their potential as non-antibiotic antimicrobial materials for biomedical applications against drug-resistant bacteria.

## 2. Results and Discussion

### 2.1. Structural and Morphological Characterization

The FeCoNiCuZn-ZnIn_2_S_4_ (HEA-ZIS) composite was successfully fabricated via a two-step strategy involving solvothermal synthesis of ZnIn_2_S_4_ (ZIS) followed by an impregnation-driven assembly with HEA (Figure 1a). Zeta potential analysis reveals that ZIS possesses a negative surface charge (−29.6 mV), which provides a favorable electrostatic driving force for the adsorption and immobilization of multimetallic HEA species (Figure S1), thereby promoting the formation of a tightly coupled heterointerface. The morphology of the samples was investigated by scanning electron microscopy (SEM). Pristine ZIS exhibits a typical hierarchical nanoflower structure assembled from ultrathin nanosheets, with an average diameter of approximately 3–5 µm (Figure 1b and Figure S2a). After incorporation of HEA, the resultant 3%HEA-ZIS (Figure 1c and Figure S2b) retains this nanoflower-like architecture without noticeable structural collapse, indicating that the loading of HEA does not disrupt the intrinsic framework of ZIS. Such structural integrity is essential for preserving high surface area and abundant accessible active sites.

Transmission electron microscopy (TEM) observations further confirm the microstructural features. Both ZIS and 3%HEA-ZIS display a three-dimensional nanosheet-assembled architecture (Figure S3), consistent with SEM results. High-resolution TEM (HRTEM) images reveal clear lattice fringes with an interplanar spacing of 0.33 nm, which corresponds to the (102) plane of hexagonal ZnIn_2_S_4_ and confirms the good crystallinity of the samples (Figure 1d). Notably, no distinct HEA nanoparticles are directly observed in the TEM images of 3%HEA–ZIS. This may be attributed to the similar atomic numbers of the FeCoNiCuZn HEA elements and the Zn-based host, resulting in limited contrast, as well as the ultrasmall size and high dispersion of the HEA species and their strong interfacial coupling with the ZnIn_2_S_4_ matrix. Nevertheless, pronounced lattice distortion and dislocations were detected, implying that the introduction of HEA induces structural perturbation (Figure 1e). Elemental mapping analysis (Figure 1f) demonstrates that S, In, Zn, Fe, Co, Ni, and Cu are uniformly distributed across the entire structure, confirming the homogeneous dispersion of HEA species on the ZIS matrix. This uniform integration is critical for maximizing interfacial contact and enabling efficient synergistic interactions within the heterostructure.

X-ray diffraction (XRD) was employed to analyze the crystallographic structure and phase composition of the samples. As shown in Figure 2a, the pristine ZIS displays distinct diffraction peaks at 21.7°, 27.7°, 30.5°, 39.8°, 47.5°, 52.5°, and 55.7°, which can be indexed to the (006), (102), (104), (108), (110), (116), and (022) planes of hexagonal ZnIn_2_S_4_, confirming the successful formation of highly crystalline ZIS (JCPDS Card No. 65-2023; Powder Diffraction File (PDF). Joint Committee on Powder Diffraction Standards (JCPDS): Swarthmore, PA, USA, 1978) [67,68,69]. Upon incorporation of HEA (1–7 wt%), the diffraction patterns of the HEA-ZIS composites remain largely unchanged, with no additional peaks attributable to metallic phases or impurities, indicating that the crystal structure of ZIS is well preserved. Notably, a gradual increase in peak intensity accompanied by slight peak broadening is observed with increasing HEA loading, reflecting modifications in diffraction features and suggesting successful integration of HEA along with increased lattice disorder.

X-ray photoelectron spectroscopy (XPS) was employed to elucidate the elemental composition and electronic states of the 3%HEA-ZIS composite. As shown in the survey spectrum (Figure 2b), Co, Ni, Cu, Zn, S, and In are simultaneously detected, confirming the successful incorporation of HEA species into the ZIS matrix. Notably, the Fe signal is not discernible in the survey spectrum due to overlap with the In 3p region and its relatively low intensity, resulting in signal masking at the full-spectrum level. High-resolution spectra further reveal the multivalent nature of the metallic components. In the Fe 2p spectrum (Figure 2c), the peaks at 709.8, 711.4, and 713.4 eV are assigned to Fe^0^ 2p_3/2_, Fe^2+^ 2p_3/2_, and Fe^3+^ 2p_3/2_, respectively, accompanied by a satellite peak at 719.1 eV [70,71]. The Co 2p spectrum (Figure 2d) shows characteristic peaks at 778.4, 780.5, and 781.7 eV, corresponding to Co^0^ 2p_3/2_, Co^3+^ 2p_3/2_, and Co^2+^ 2p_3/2_ [70,72]. Similarly, the Ni 2p spectrum (Figure 2e) reveals the coexistence of multiple oxidation states, with peaks at 852.4, 854.8, and 856.2 eV assigned to the 2p_3/2_ levels of Ni^0^, Ni^2+^, and Ni^3+^, respectively [70]. In contrast, the Cu 2p spectrum (Figure 2f) exhibits dominant metallic Cu^0^ signals at 932.1 eV (2p_3/2_) and 952.1 eV (2p_1/2_), without obvious satellite features [73]. In addition, the characteristic peaks of Zn^2+^ at 1022.1 eV (Zn 2p_3/2_) and 1045.1 eV (Zn 2p_1/2_), In^3+^ at 444.9 eV (In 3d_5/2_) and 452.5 eV (In 3d_3/2_), as well as S^2−^ at 161.6 eV (S 2p_3/2_) and 162.8 eV (S 2p_1/2_) are clearly observed (Figure S4), confirming the preserved chemical states of the ZIS framework [38,73,74]. Notably, compared with standard references [75,76], the binding energies of Fe^0^ and Co^0^ exhibit positive shifts, whereas those of Ni^0^ and Cu^0^ shift toward lower energies (Figure S5), indicating pronounced electron redistribution among the multimetallic components. This charge transfer behavior is attributed to the differences in electronegativity (Fe < Co < Ni < Cu), suggesting strong electronic coupling within the HEA system and at the 3%HEA-ZIS interface.

### 2.2. Photocatalytic Antibacterial Performance

The photocatalytic antibacterial activity of HEA-ZIS was systematically evaluated using methicillin-resistant *Staphylococcus aureus* (MRSA). To optimize the composition of the high-entropy alloy, a series of composites with varying HEA contents (1%, 3%, 5%, and 7%) was prepared and assessed (Table S1). MRSA suspensions (1 × 10^4^ CFU mL^−1^) were incubated with different samples (0.5 mg mL^−1^) for 1 h, followed by 30 min of simulated solar irradiation and an additional 4 h of incubation. Quantitative colony counting revealed bacterial survival rates of 43.81 ± 1.10%, 14.83 ± 2.11%, 35.40 ± 2.62%, and 60.80 ± 9.60% for 1%, 3%, 5%, and 7%HEA-ZIS, respectively, with 3%HEA-ZIS exhibiting the most potent antibacterial activity (Figure S6a,b). The 5–7% HEA loading resulted in poorer antibacterial performance, likely because excessive HEA introduces charge recombination centers, surface shielding, and particle aggregation, which may impede charge separation, light utilization, and active site accessibility, thus suppressing ROS generation [77,78,79]. Accordingly, 3%HEA-ZIS was selected for subsequent investigations.

To further quantify the antibacterial potency of 3%HEA-ZIS, MRSA suspensions were exposed to varying concentrations of the composite under visible-light irradiation for 30 min, followed by incubation at 37 °C for 12 h. The minimum inhibitory concentration (MIC) and minimum bactericidal concentration (MBC) were determined based on OD_600_ measurements and colony counting assays (Figure S7a). Notably, at a concentration of 0.2 mg mL^−1^, the bacterial survival rate decreased to 8.49 ± 0.66%, corresponding to a 3.50 ± 0.28 log_10_(CFU mL^−1^) reduction (Figure S7b,c). The MIC and MBC were identified as 0.20 and 0.30 mg mL^−1^, respectively, demonstrating the strong antibacterial efficacy of 3%HEA-ZIS.

Subsequently, the antibacterial performance of pristine ZIS and 3%HEA-ZIS was comparatively evaluated. MRSA suspensions were incubated with 0.30 mg mL^−1^ of ZIS or 3%HEA-ZIS for 1 h, followed by 30 min visible-light irradiation and 24 h incubation in the dark at 37 °C. Under dark conditions, no significant differences in bacterial survival were observed among PBS, ZIS, and 3%HEA-ZIS groups, indicating negligible intrinsic antibacterial activity. In contrast, under visible-light irradiation, pronounced differences emerged. The bacterial survival rates for PBS, ZIS, and 3%HEA-ZIS were 100.02 ± 0.58%, 11.22 ± 1.86%, and 1.69 ± 0.74%, respectively (Figure 3a–c). Plate counting further confirmed that ZIS and 3%HEA-ZIS induced bacterial reductions of 4.03 ± 0.09 and 5.91 ± 0.28 log_10_(CFU mL^−1^), respectively. Live/dead staining assays (Figure 3d,e and Figure S8) corroborated these findings, showing a markedly higher proportion of dead bacteria (red fluorescence) in the 3%HEA-ZIS + light group (89.56 ± 4.73%) compared to the ZIS + light group (56.88 ± 0.84%). The enhanced antibacterial performance of 3%HEA-ZIS compared to ZIS may be attributed to the synergistic effects of HEA incorporation, which likely promotes charge separation and interfacial electron transfer while providing abundant active sites, thereby enhancing ROS generation.

Collectively, these results indicate that 3%HEA-ZIS enables highly efficient MRSA eradication under visible-light irradiation, highlighting its strong potential for antibacterial applications.

### 2.3. In Vitro Antibiofilm Activity and Biocompatibility Evaluation

Given that biofilm formation markedly enhances bacterial resistance to antimicrobial therapies, evaluating antibiofilm performance is essential for assessing the practical applicability of antibacterial materials. Accordingly, the antibiofilm activity of 3%HEA-ZIS was systematically investigated. MRSA suspensions (10^6^ CFU mL^−1^) pretreated with ZIS or 3%HEA-ZIS were cultured in 48-well plates for 48 h, and biofilm formation was quantified via crystal violet staining (Figure 4a,b and Figure S9a). Under dark conditions, no significant inhibition of biofilm formation was observed across all groups, indicating that the material alone is insufficient to disrupt biofilm formation. In contrast, upon visible-light irradiation, 3%HEA-ZIS exhibited markedly enhanced antibiofilm activity, achieving an 84.34% ± 3.92% reduction in biofilm biomass relative to the control group, with only sparse residual biofilm attachment remaining on the substrate surface. Notably, this inhibition efficiency was substantially higher than that of pristine ZIS under identical irradiation conditions (62.21% ± 4.00%), demonstrating the superior capability of 3%HEA-ZIS to suppress MRSA biofilm formation and disrupt biofilm integrity.

To further elucidate the spatial disruption and structural integrity of biofilms beyond bulk biomass quantification, confocal laser scanning microscopy (CLSM) combined with SYTO9 staining was employed to reconstruct the three-dimensional architecture of the biofilms (Figure 4c,d and Figure S9b). In the absence of light, all groups retained highly compact and continuous biofilm structures with thicknesses exceeding 30 µm. Upon visible-light irradiation, the ZIS-treated group exhibited evident structural disruption, characterized by the emergence of discontinuities within the biofilm matrix, a reduction in biofilm thickness (15.9 ± 1.27 μm), and decreased coverage of viable cells (20.78 ± 6.40%). In contrast, the 3%HEA-ZIS-treated group showed a far more pronounced destructive effect, with the biofilm thickness reduced to below 10 μm and the residual structure becoming highly sparse and fragmented, accompanied by only minimal coverage of viable cells (3.29 ± 2.29%). The enhanced biofilm disruption capability of 3%HEA-ZIS may be attributed to its superior ROS generation and improved penetration into the biofilm matrix, enabling more efficient oxidative damage throughout the entire biofilm. Overall, these results demonstrate that 3%HEA–ZIS exhibits efficient photocatalytic antibiofilm activity under visible-light irradiation, suggesting its potential for combating drug-resistant bacteria and biofilm-associated infections.

Considering that effective antibacterial materials for biomedical applications must combine high antimicrobial efficacy with acceptable biocompatibility, the in vitro biosafety of ZIS and 3%HEA–ZIS was subsequently evaluated using mouse fibroblast L929 cells. According to the CCK-8 assay, both materials exhibited minimal cytotoxicity, with cell viability remaining above 90% even at a concentration as high as 400 μg mL^−1^ after 24 h of incubation (Figure S10a,b). This result indicates that neither pristine ZIS nor HEA-modified ZIS induces significant inhibition under the tested conditions. Consistently, live/dead fluorescence staining further confirmed the favorable cytocompatibility of the materials. After 24 h of co-incubation, L929 cells maintained normal spindle-like morphology with well-spread adhesion, and negligible red fluorescence (dead cells) was observed, while the majority of cells exhibited strong green fluorescence, indicative of intact cell membranes and high viability (Figure S10c). These results collectively demonstrate that both ZIS and 3%HEA–ZIS possess good in vitro biocompatibility.

### 2.4. Photocatalytic Mechanism Investigation

Given the pronounced antibacterial and antibiofilm activity of 3%HEA-ZIS, its photocatalytic mechanism was further investigated with a focus on reactive oxygen species (ROS), which are recognized as key mediators in photocatalytic antimicrobial processes [80]. In such systems, ROS generation proceeds through sequential processes of light absorption, charge separation and migration, reactant adsorption/activation (O_2_ and H_2_O), and subsequent interfacial redox reactions producing •O_2_^−^, •OH, and ^1^O_2_ [81,82]. Accordingly, the optical properties, band structure, charge transfer behavior, and surface physicochemical characteristics were systematically investigated, as these factors collectively dictate ROS generation and the resulting antibacterial and antibiofilm performance. 

The light-harvesting properties were first evaluated by UV-vis diffuse reflectance spectroscopy (DRS) (Figure 5a). Pristine ZIS exhibits an absorption edge at ~500 nm, corresponding to its intrinsic band gap. Upon HEA incorporation, the visible-light absorption (600–800 nm) is enhanced, accompanied by a slight red shift, indicating modulation of the electronic structure. Specifically, higher HEA loadings (5% and 7%) result in elevated baseline absorption, which is likely associated with light scattering or metal-related absorption rather than effective band-to-band transitions [83,84,85]. Tauc plot analysis (Figure 5b) reveals a gradual narrowing of the band gap from 2.51 eV (ZIS) to 2.48 eV (3%HEA-ZIS) and further to 2.42 eV at higher loadings, suggesting the introduction of electronic interactions. While these changes improve light absorption, the optimal 3%HEA-ZIS may achieve a balance between enhanced photon harvesting and suppressed non-radiative losses, consistent with its superior antibacterial performance, whereas excessive loading may induce recombination or optical shielding effects, limiting the effective utilization of photogenerated carriers [65,86].

To further evaluate the thermodynamic feasibility of ROS generation, the band structure was determined through Mott–Schottky and XPS valence band (VB) analyses (Figure 5c–f). The flat-band potentials derived from the Mott–Schottky plots indicate that the conduction band (CB) positions of ZIS and 3%HEA-ZIS are approximately −1.03 V vs. Ag/AgCl and −0.94 V vs. Ag/AgCl, respectively. After conversion to the normal hydrogen electrode (NHE) scale, the CB potentials are estimated to be −0.93 V vs. NHE (ZIS) and −0.84 V vs. NHE (3%HEA-ZIS). Both values are more negative than the redox potential of O_2_/•O_2_^−^ (−0.33 V vs. NHE) [23], demonstrating that photogenerated electrons can thermodynamically reduce dissolved O_2_ to •O_2_^−^. Based on the band gap values, the VB potentials are calculated to be 1.58 V vs. NHE (ZIS) and 1.64 V vs. NHE (3%HEA-ZIS), which are in good agreement with the VB positions determined from XPS VB spectra (1.62 and 1.69 eV, respectively; Figure 5e). These VB potentials are sufficiently positive to drive the stepwise reduction in O_2_ to H_2_O_2_ (0.68 V vs. NHE) and subsequently to •OH, thereby providing the necessary driving force for ROS generation [87]. The resulting band structure (Figure 5f) indicates that HEA incorporation induces a slight shift in both CB and VB positions while preserving suitable redox potentials for ROS production. This optimized band alignment ensures efficient participation of photogenerated charge carriers in interfacial redox reactions, thereby facilitating the formation of multiple ROS and contributing to the enhanced photocatalytic antibacterial and antibiofilm performance.

Building on the favorable band structure, the charge separation and transfer behavior were further investigated to clarify the kinetic factors governing ROS generation. Transient photocurrent measurements (IT) reveal that 3%HEA-ZIS exhibits a significantly enhanced photocurrent response, which is approximately 4.35 times higher than that of pristine ZIS, indicating markedly improved separation efficiency of photogenerated electron-hole pairs (Figure 6a). Consistently, electrochemical impedance spectroscopy (EIS) shows a reduced Nyquist semicircle radius for 3%HEA-ZIS, corresponding to a lower charge transfer resistance, while cyclic voltammetry (CV) curves display increased redox current density (Figure 6b,c). These results collectively demonstrate that HEA incorporation promotes interfacial charge transfer and suppresses charge recombination, ensuring that more charge carriers participate in surface redox reactions.

Given that the photocatalytic eradication of bacteria is fundamentally governed by the generation of ROS at the catalyst–reactant (O_2_ and H_2_O) interface, optimizing the surface physicochemical properties is paramount. The water contact angle decreases from 50.15° for ZIS to 46.61° for 3%HEA-ZIS, indicating enhanced hydrophilicity that promotes a more intimate interaction between the catalyst and the aqueous reaction substrates. To further probe surface reactivity, oxygen temperature-programmed desorption (O_2_-TPD) analysis was conducted. The profiles show that pure ZIS exhibits weak low-temperature desorption, a moderate peak at ~300 °C, and negligible high-temperature signals, whereas 3%HEA-ZIS displays markedly enhanced and broadened desorption with a pronounced high-temperature tail, evidencing a substantial increase in reactive chemisorbed oxygen species. Collectively, these results demonstrate that HEA incorporation synergistically optimizes interfacial affinity and oxygen activation capability, thereby providing a favorable platform for efficient ROS generation and enhanced photocatalytic performance.

To elucidate the evolution of reactive oxygen species during photocatalysis, in situ diffuse reflectance infrared Fourier transform spectroscopy (in situ DRIFTS) measurements were performed under continuous light irradiation (Figure 7c,d and Figure S11). Specifically, the bands at 950–1000 cm^−1^ are assigned to surface-bound •O_2_^−^, indicating efficient electron transfer to adsorbed O_2_ [88,89]. With prolonged irradiation, bands in the 1120–1200 cm^−1^ range appear, corresponding to •OOH species or H_2_O_2_, suggesting the involvement of proton-coupled electron transfer and peroxide formation [89,90]. Meanwhile, the distinct feature at ~1044 cm^−1^ is attributed to a 1,4-endoperoxide species (-O-O-), characteristic of Yeager-type O_2_ adsorption [91]. The emergence of a band at ~877 cm^−1^ is assigned to hydroxyl-related species (•OH/-OH), likely originating from the decomposition of peroxide intermediates and/or surface water oxidation processes [92]. The progressive intensification of these bands with irradiation time confirms the continuous generation and accumulation of ROS. Notably, HEA-ZIS exhibits more pronounced spectral evolution than pristine ZIS, indicating enhanced interfacial charge transfer and O_2_ activation capability. Collectively, these results suggest a cascade oxygen activation pathway involving O_2_ → •O_2_^−^ → •OOH/H_2_O_2_ intermediates, followed by the formation of •OH species, indicating multiple ROS-generating routes operating synergistically under photocatalytic conditions.

To provide additional support for these findings, electron paramagnetic resonance (EPR) spectroscopy was conducted to directly probe reactive oxygen species and evaluate the redox capability. Upon simulated sunlight irradiation, •O_2_^−^ and •OH were successfully captured by 5,5-dimethyl-1-pyrroline N-oxide (DMPO) in methanol and aqueous media, respectively. As shown in Figure 7e,f, 3%HEA-ZIS exhibits significantly stronger DMPO-•O_2_^−^ and DMPO-•OH signals than pristine ZIS, confirming its superior ROS generation capacity. These observations, consistent with the DRIFTS results, collectively demonstrate that HEA incorporation effectively enhances charge separation and promotes oxygen activation, thereby boosting ROS production.

In summary, the above results demonstrate that 3%HEA-ZIS achieves a synergistic optimization of light absorption, band structure, charge carrier dynamics, and interfacial properties, collectively enabling efficient generation of ROS. The incorporation of HEA not only enhances visible-light harvesting and slightly narrows the band gap, but also tailors the band alignment to favor the thermodynamically feasible reduction in O_2_ to •O_2_^−^ and its subsequent transformation. Meanwhile, the significantly improved charge separation and transfer efficiency suppresses electron-hole recombination, allowing more photogenerated carriers to participate in interfacial redox reactions. In addition, the enhanced surface hydrophilicity and enriched chemisorbed oxygen species further promote interfacial oxygen activation. Combined in situ DRIFTS and EPR analyses reveal a cascade ROS generation pathway of O_2_ → •O_2_^−^ → •OOH/H_2_O_2_ → •OH, with multiple ROS operating synergistically. These structure-property-mechanism correlations collectively account for the superior antibacterial and antibiofilm performance of 3%HEA-ZIS.

## 3. Materials and Methods

### 3.1. Materials and Reagents

Indium (III) chloride tetrahydrate (InCl_3_·4H_2_O), absolute ethanol, Iron(III) acetylacetonate, Cobalt(III) acetylacetonate, Nickel(II) acetylacetonate, Copper(II) acetylacetonate, Zinc(II) acetylacetonate, ethylene glycol and acetone were purchased from Sigma-Aldrich (St. Louis, MO, USA). Zinc chloride (ZnCl_2_) and thioacetamide (TAA) were purchased from China National Medicines Corporation Ltd. (Beijing, China). LIVE/DEAD BacLight Bacterial Viability Kit (L7012) was purchased from ThermoFisher Scientific (Waltham, MA, USA). The 0.1% crystal violet staining solution was purchased from Beyotime. A live/dead cell staining kit (Calcein AM/PI) was obtained from Solarbio (Beijing, China). The CCK-8 assay kit was supplied by the Beyotime Institute of Biotechnology (Shanghai, China).

### 3.2. Synthesis of ZnIn_2_S_4_ (ZIS)

ZnIn_2_S_4_ was synthesized via a modified solvothermal approach [93]. Specifically, ZnCl_2_ (0.4 mmol), InCl_3_·4H_2_O (0.8 mmol), and thioacetamide (TAA, 3.2 mmol) were dissolved in 40 mL of a mixed solvent containing ethanol and deionized water (1:1, *v*/*v*). The solution was sonicated for 10 min and subsequently stirred at 800 rpm for 30 min to obtain a homogeneous precursor. The resulting mixture was transferred into a 100 mL Teflon-lined stainless-steel autoclave and heated at 180 °C for 24 h. After naturally cooling to room temperature, the precipitate was collected by centrifugation and washed several times with an ethanol/water mixture (1:1, *v*/*v*) at 8000 rpm for 5 min per cycle. The final product was dried at 60 °C overnight to obtain ZnIn_2_S_4_ powder.

### 3.3. Synthesis of FeCoNiCuZn High-Entropy Alloy (HEA)

The FeCoNiCuZn high-entropy alloy was prepared through a solvothermal route [94]. Metal precursors, including iron(III) acetylacetonate, cobalt(III) acetylacetonate, nickel(II) acetylacetonate, copper(II) acetylacetonate, and zinc(II) acetylacetonate, were dissolved in a mixed solvent of acetone and ethanol (1:1, *v*/*v*; 30 mL total volume), with each metal species at a concentration of 5 × 10^−3^ M. The precursor solution was then transferred into a Teflon-lined autoclave and maintained at 200 °C for 24 h. After cooling to room temperature, the solid product was separated by centrifugation (8000 rpm, 10 min), washed twice with acetone to remove residual organics, and dried overnight to yield the HEA powder.

### 3.4. Preparation of FeCoNiCuZn-ZnIn_2_S_4_ (HEA-ZIS) Composites

The HEA-ZIS composites were fabricated via the impregnation method. Typically, 500 mg of ZIS was dispersed in a mixed solvent composed of ethanol (15 mL), deionized water (15 mL), and ethylene glycol (10 mL), followed by stirring for 30 min to ensure uniform dispersion. Subsequently, a designated amount of HEA powder (1–7 wt%) was introduced into the suspension, and the mixture was continuously stirred at 1000 rpm for 24 h to promote intimate contact between components. The suspension was then allowed to stand for 24 h to facilitate sedimentation. The collected solids were washed repeatedly with an ethanol/water mixture (1:1, *v*/*v*) via centrifugation (8000 rpm, 5 min per cycle) and finally dried at 60 °C overnight. A series of HEA-ZIS composites with varying HEA loadings was thus obtained.

### 3.5. Material Characterization

The morphology and elemental distribution of the samples were characterized by field-emission scanning electron microscopy (FESEM, Thermo Scientific Apreo ChemiSEM, Waltham, MA, USA) and high-resolution transmission electron microscopy (HRTEM, FEI Tecnai G2 F20, FEI Company, Hillsboro, OR, USA) equipped with an energy-dispersive X-ray spectroscopy (EDS) system. The crystal structure was examined using powder X-ray diffraction (PXRD, Bruker D8 Advance, Bruker AXS GmbH, Karlsruhe, Germany). X-ray photoelectron spectroscopy (XPS, Thermo Fisher EscaLab 250Xi, Thermo Fisher Scientific, Waltham, MA, USA) was employed to analyze the surface chemical states. Mott–Schottky measurements were conducted in a 3.0 M KCl electrolyte using a CHI 660E electrochemical workstation (Shanghai Chenhua Instrument Co., Ltd., Shanghai, China). The optical absorption properties were evaluated by UV-vis diffuse reflectance spectroscopy (UV-DRS, PerkinElmer LAMBDA 1050+, PerkinElmer, Waltham, MA, USA). The specific surface area was determined through Brunauer–Emmett–Teller (BET) analysis on a Matrix1000 instrument (Beijing JWGB Instruments Co., Ltd., Beijing, China). Electron paramagnetic resonance (EPR, Bruker A300-10/12, Bruker BioSpin GmbH, Karlsruhe, Germany) spectroscopy was utilized to monitor the generation of reactive radical species. The elemental composition of the specimens was precisely quantified using inductively coupled plasma optical emission spectrometry (ICP-OES, Analytik Jena PQ9000, Analytik Jena GmbH, Jena, Germany). Bacterial fluorescence and spatial biofilm distribution were characterized using a Zeiss LSM980 confocal laser scanning microscope (Carl Zeiss Microscopy GmbH, Jena, Germany). Simulated solar irradiation was provided by an MC-PF300C xenon lamp (Beijing Merry Change Technology Co., Ltd., Beijing, China) equipped with a visible-light cut-off filter.

### 3.6. In Situ DRIFTS Measurements

To probe the evolution of surface oxygen-containing intermediates during the reaction, in situ diffuse reflectance infrared Fourier transform spectroscopy (DRIFTS) was performed on a Bruker INVENIO-S FTIR spectrometer (Bruker Optics GmbH, Ettlingen, Germany) equipped with a Harrick diffuse reflectance accessory (Harrick Scientific Products, Inc., Pleasantville, NY, USA). A 10 mg portion of the sample was evenly laid out in the holder, followed by pressing to yield a flat and even surface. Before data acquisition, the system was purged with Ar to obtain a stable background spectrum. Afterwards, O_2_ was bubbled through water and then fed into the reaction chamber until the adsorption equilibrium was attained. A 300 W xenon lamp was subsequently turned on to start the photocatalytic reaction. In situ FTIR spectra were recorded over a period of 0–50 min.

### 3.7. Antibacterial Assays

Methicillin-resistant *Staphylococcus aureus* (MRSA, ATCC 43300) was employed as the model microorganism for antibacterial evaluation. Bacteria were revived from −80 °C stocks, streaked on Tryptic Soy Agar (TSA) plates, and subcultured before use. A single colony was inoculated into TSA broth and cultured at 37 °C (200 rpm) to the mid-log phase (OD_600_ ≈ 0.4–0.5), then diluted to 10^4–^10^6^ CFU mL^−1^. For antibacterial evaluation, 3 mL of bacterial suspension was mixed with 3 mL of sample dispersion in a sterile glass tube and incubated in the dark for 1 h. The mixture was then irradiated under simulated sunlight (100 mW cm^−2^, λ > 420 nm) with stirring (120 rpm) for 30 min or kept in the dark. After treatment, samples were incubated at 37 °C for 12–24 h before analysis.

### 3.8. Determination of MIC and MBC

The minimum inhibitory concentration (MIC) and minimum bactericidal concentration (MBC) of the HEA-ZIS materials were evaluated against MRSA. Briefly, bacterial suspensions were exposed to a dose-dependent series of HEA-ZIS (0, 0.05, 0.1, 0.2, 0.3, and 0.4 mg/mL) under simulated light for 30 min, followed by incubation at 37 °C overnight. Bacterial growth was evaluated by measuring the optical density at 600 nm (OD_600_). Aliquots were then serially diluted, plated on agar, and incubated for colony counting. Herein, the MIC is defined as the lowest concentration that inhibits ≥ 90% of visible bacterial growth, while the MBC represents the threshold concentration required to achieve a ≥99% reduction in the initial bacterial population.

### 3.9. Live/Dead Staining

The treated bacterial cells were washed twice with phosphate-buffered saline (PBS) and resuspended in 1 mL of PBS. SYTO9 and propidium iodide (PI) (1.5 µL each) were then added, followed by incubation at 37 °C for 30 min in the dark. After staining, 20 µL of the suspension was deposited onto a clean glass slide for confocal laser scanning microscopy observation. Fluorescence images were acquired using excitation wavelengths of 488 nm and 555 nm, corresponding to live (green) and dead (red) bacteria, respectively. Quantitative analysis was performed using ImageJ software (version 1.43u, National Institutes of Health, Bethesda, MD, USA) by counting green- and red-fluorescent cells.

### 3.10. Anti-Biofilm Activity Assessment

The antibiofilm performance was evaluated using crystal violet staining and SYTO9 fluorescence analysis.

For crystal violet staining, bacterial suspensions (1 mL, 10^6^ CFU mL^−1^) subjected to different treatments were transferred into 48-well plates and incubated at 37 °C for 48 h, with fresh trypsin soybean broth replaced daily. After incubation, non-adherent cells were removed, and the attached biofilms were fixed with 95% methanol for 1 h. The samples were then stained with 0.1% crystal violet for 30 min. Excess dye was eliminated with deionized water, and the retained stain was dissolved in 33% glacial acetic acid. The absorbance at 590 nm (OD_590_) was recorded using a microplate reader. The Biofilm retention rate was calculated as:

Biofilm retention rate (%) = OD _sample_/OD _control_ × 100%

where OD _control_ corresponds to the PBS-treated group in the dark, and OD _sample_ represents the values obtained from different treatment groups under light or dark conditions.

For biofilm viability analysis, sterile coverslips were placed in 6-well plates prior to use. Treated bacterial suspensions (4 mL, 10^6^ CFU mL^−1^) were added and cultured at 37 °C for 2 days with daily medium renewal to allow biofilm formation on the coverslip surface. After gently washing with PBS to remove planktonic bacteria, the established biofilms were stained with SYTO9 (5 μM in PBS) for 30 min in the dark. Z-stack images were obtained by confocal microscopy, followed by 3D reconstruction using ZEN and quantitative analysis of viable biofilm coverage with Imaris.

### 3.11. In Vitro Biocompatibility Evaluation

The in vitro biocompatibility of the as-prepared materials was assessed by evaluating cytotoxicity toward mouse fibroblast L929 cells (American Type Culture Collection, ATCC, Manassas, VA, USA) using both the Cell Counting Kit-8 (CCK-8) assay (Beyotime Institute of Biotechnology, Shanghai, China) and live/dead fluorescence staining.

For the CCK-8 assay, L929 cells were seeded in 96-well plates at a density of 4 × 10^3^ cells per well and cultured in Dulbecco’s Modified Eagle Medium (DMEM, high glucose) supplemented with 10% fetal bovine serum and 1% penicillin/streptomycin for 24 h. The culture medium was then replaced with 100 μL of fresh DMEM containing dispersed materials at designated concentrations, followed by incubation for an additional 24 h. After treatment, the medium was substituted with 100 μL of a CCK-8 working solution (CCK-8 reagent/DMEM = 1:9, *v*/*v*) and incubated for 2 h. The absorbance at 450 nm was subsequently recorded using a microplate reader. Cell viability was calculated according to the following equation:

Cell viability (%) = (OD _sample_/OD _control_) × 100%

where OD _sample_ and OD _control_ represent the absorbance values obtained from material-treated and untreated (pure DMEM) groups, respectively.

For live/dead staining, L929 cells were seeded into confocal culture dishes at a density of 8 × 10^4^ cells per dish and allowed to adhere for 24 h. After 24 h of exposure to the materials, the medium was removed and replaced with 1 mL of staining solution. Following incubation in the dark for 20 min, the stained cells were imaged using a confocal laser scanning microscope.

### 3.12. Statistical Analysis

Quantitative results are presented as the mean values accompanied by standard deviations, derived from at least two independent biological replicates. Statistical significance between groups was evaluated using either a two-tailed Student’s *t*-test or one-way analysis of variance (ANOVA), performed with GraphPad Prism (version 10.1.2.324). A significance level of *p* < 0.05 was adopted throughout the study. Statistical significance is indicated as follows: * *p* < 0.05, ** *p* < 0.01, *** *p* < 0.001, and **** *p* < 0.0001, while “ns” denotes no statistically significant difference.

## 4. Conclusions

In this work, a high-entropy alloy FeCoNiCuZn-decorated ZnIn_2_S_4_ (HEA-ZIS) heterostructure was developed as a visible-light-driven nano-antibacterial platform. Comprehensive characterizations, including SEM/TEM, XRD, and XPS, confirm that HEA incorporation preserves the hierarchical nanoflower architecture of ZIS while inducing lattice distortion, multivalent metal states, and strong interfacial electronic coupling. These structural perturbations and electronic redistributions provide abundant active sites and promote efficient charge carrier modulation. As a result, the optimized 3%HEA-ZIS exhibits outstanding antibacterial performance against MRSA under visible-light irradiation, achieving a bacterial inhibition rate of ~98% and a 5.91 log reduction, along with an MBC of 0.30 mg mL^−1^. Meanwhile, it demonstrates remarkable antibiofilm capability, removing over 84% of biofilm biomass and effectively disrupting biofilm architecture, while retaining favorable cytocompatibility toward fibroblasts in vitro. The strong antibacterial performance originates from a synergistic cascade of ROS generation promoted by HEA incorporation. The multimetallic HEA interface narrows the band gap to enhance visible-light harvesting, substantially facilitates charge separation and suppresses electron–hole recombination, and enriches surface chemisorbed oxygen to activate O_2_ more effectively. Combined in situ DRIFTS and EPR analyses reveal a stepwise ROS generation pathway O_2_ → •O_2_^−^ → •OOH/H_2_O_2_ → •OH, where multiple radical species act in concert to drive irreversible oxidative damage to bacterial cells and biofilm matrices. These in vitro findings establish a structure–property–mechanism correlation in which high-entropy alloy engineering optimizes the optical, electronic, and surface characteristics of semiconductor-based nanoantimicrobials, showing the potential of HEA-ZIS heterostructures as a biocompatible candidate for combating drug-resistant bacterial infections and biofilm-associated challenges.

## Figures and Tables

**Figure 1:**
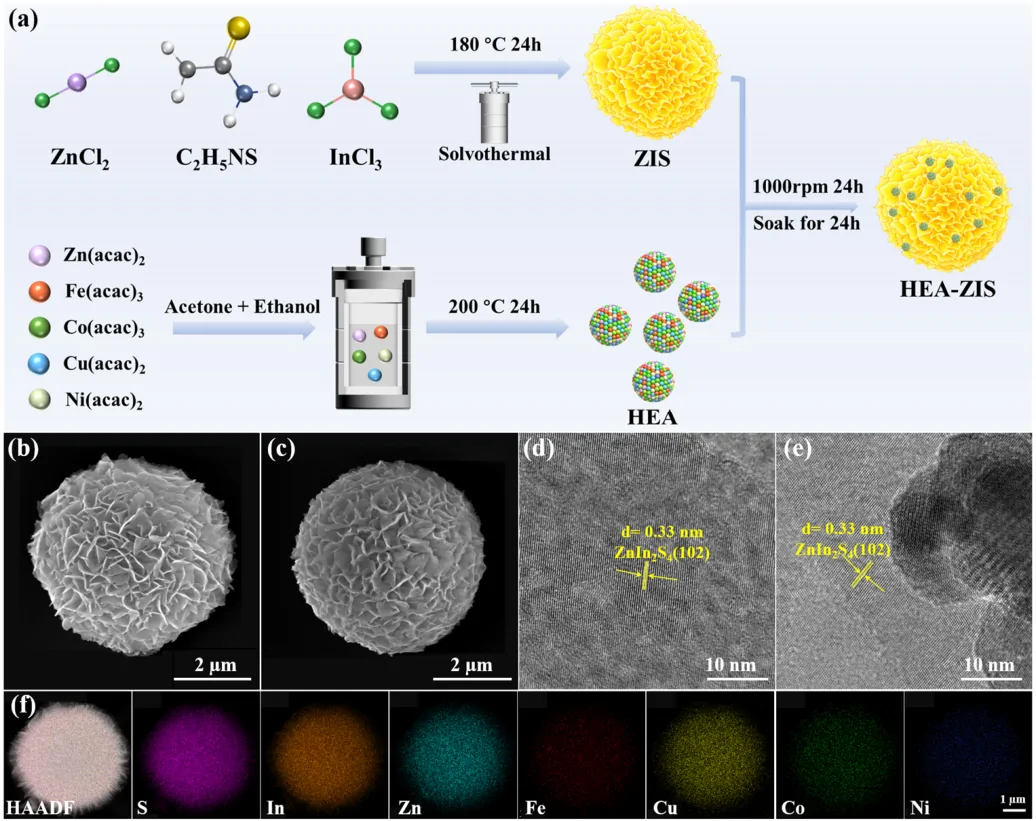
(**a**) Schematic illustration of the synthesis process of HEA-ZIS; (**b**) FESEM image of ZIS; (**c**) FESEM image of 3%HEA-ZIS; (**d**) TEM image of ZIS; (**e**) TEM image of 3%HEA-ZIS; (**f**) Energy-dispersive X-ray spectroscopy (EDS) image and corresponding elemental mapping of S, In, Zn, Fe, Cu, Co, and Ni for 3%HEA-ZIS.

**Figure 2:**
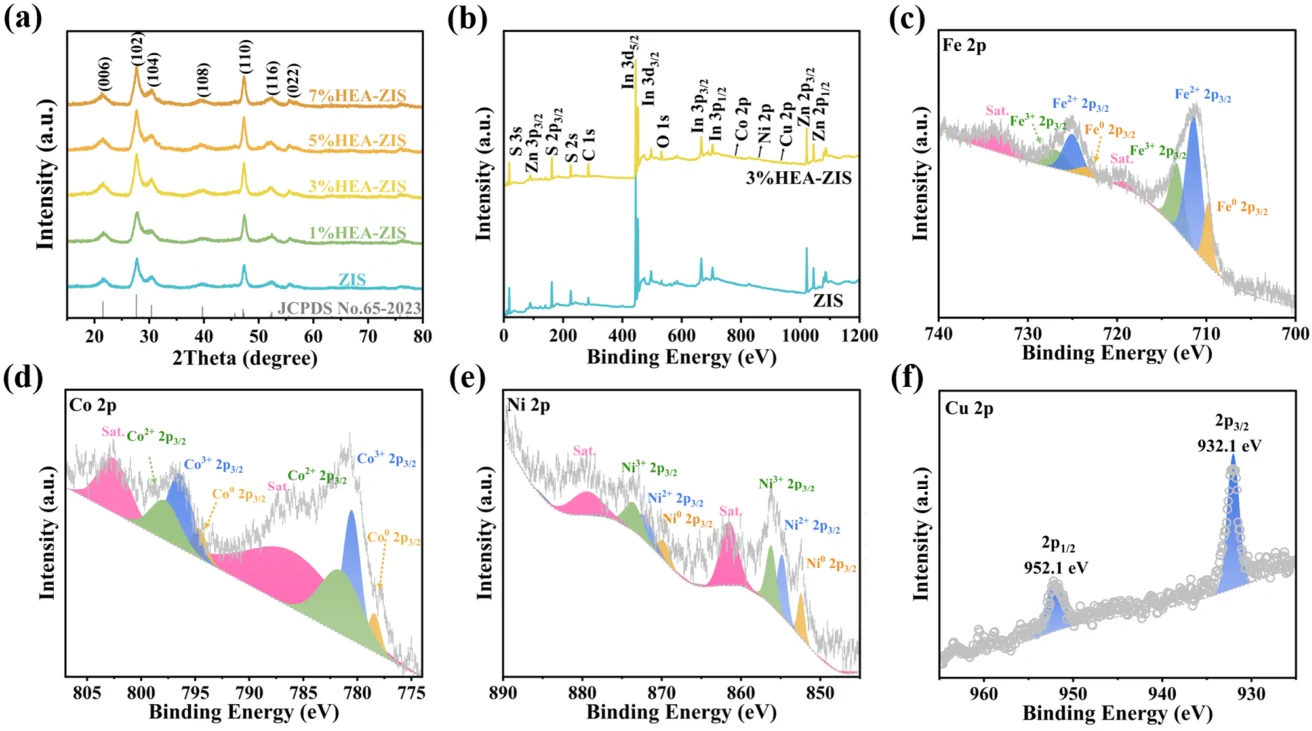
(**a**) XRD patterns of the as-prepared samples with reference to JCPDS Card No. 65-2023 (Powder Diffraction File, Joint Committee on Powder Diffraction Standards (JCPDS): Swarthmore, PA, USA, 1978) [69,70,71]; (**b**) XPS survey spectra of ZIS and 3%HEA-ZIS; (**c**–**f**) high-resolution XPS spectra of 3%HEA-ZIS: (**c**) Fe, (**d**) Co, (**e**) Ni, (**f**) Cu.

**Figure 3:**
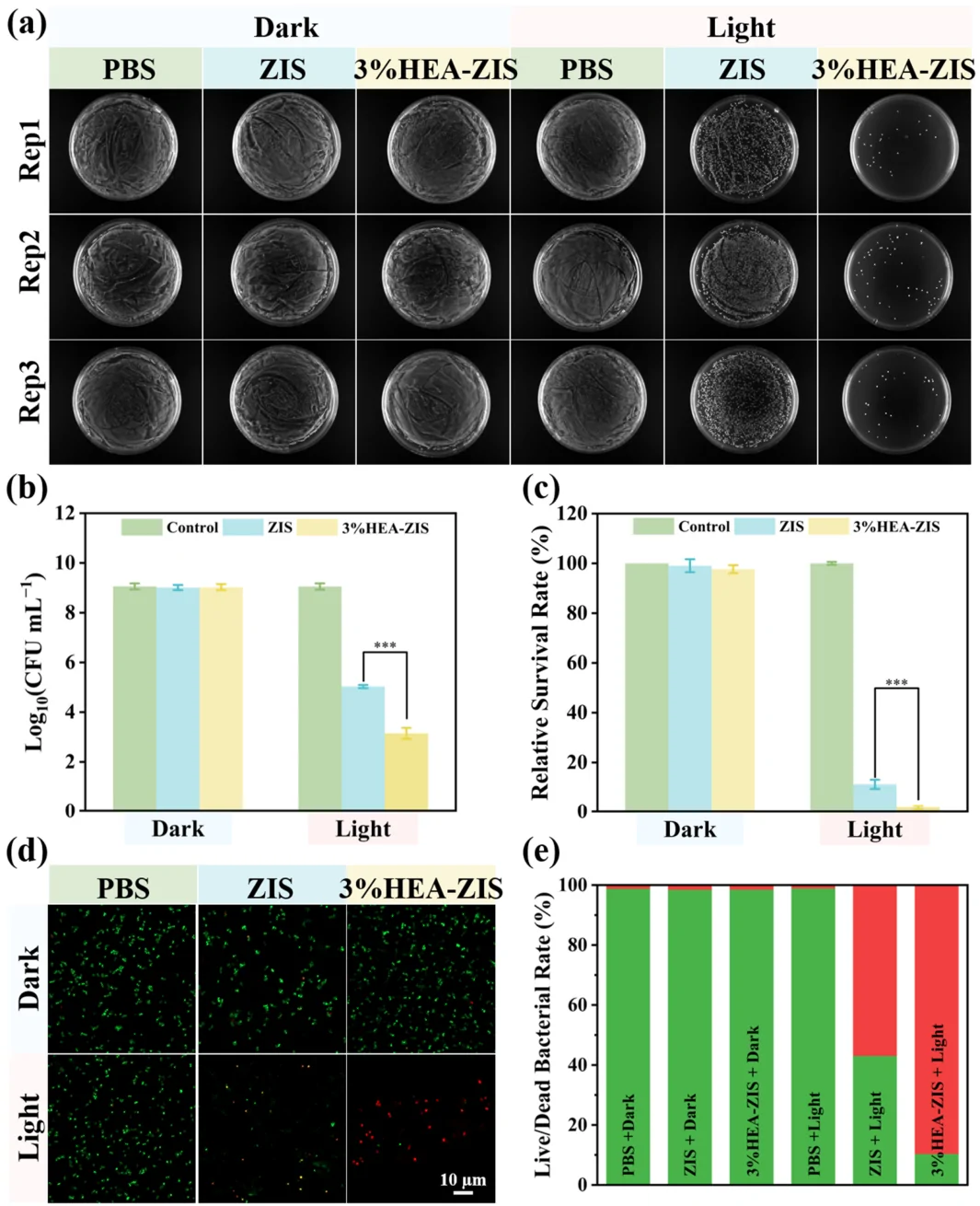
Evaluation of the antibacterial activity against MRSA. (**a**) Representative photographs of MRSA colonies on tryptic soy agar (TSA) plates post-various treatments. (**b**) Statistical analysis of bacterial concentrations expressed as Log_10_(CFU mL^−1^). *n* = 3, *** *p* < 0.001. (**c**) Relative survival rate based on OD_600_ measurements. *n* = 3, *** *p* < 0.001. (**d**) Confocal laser scanning microscopy images of bacteria stained with live/dead assays (red: dead, green: live). (**e**) Corresponding percentages of live and dead bacteria. *n* = 2.

**Figure 4:**
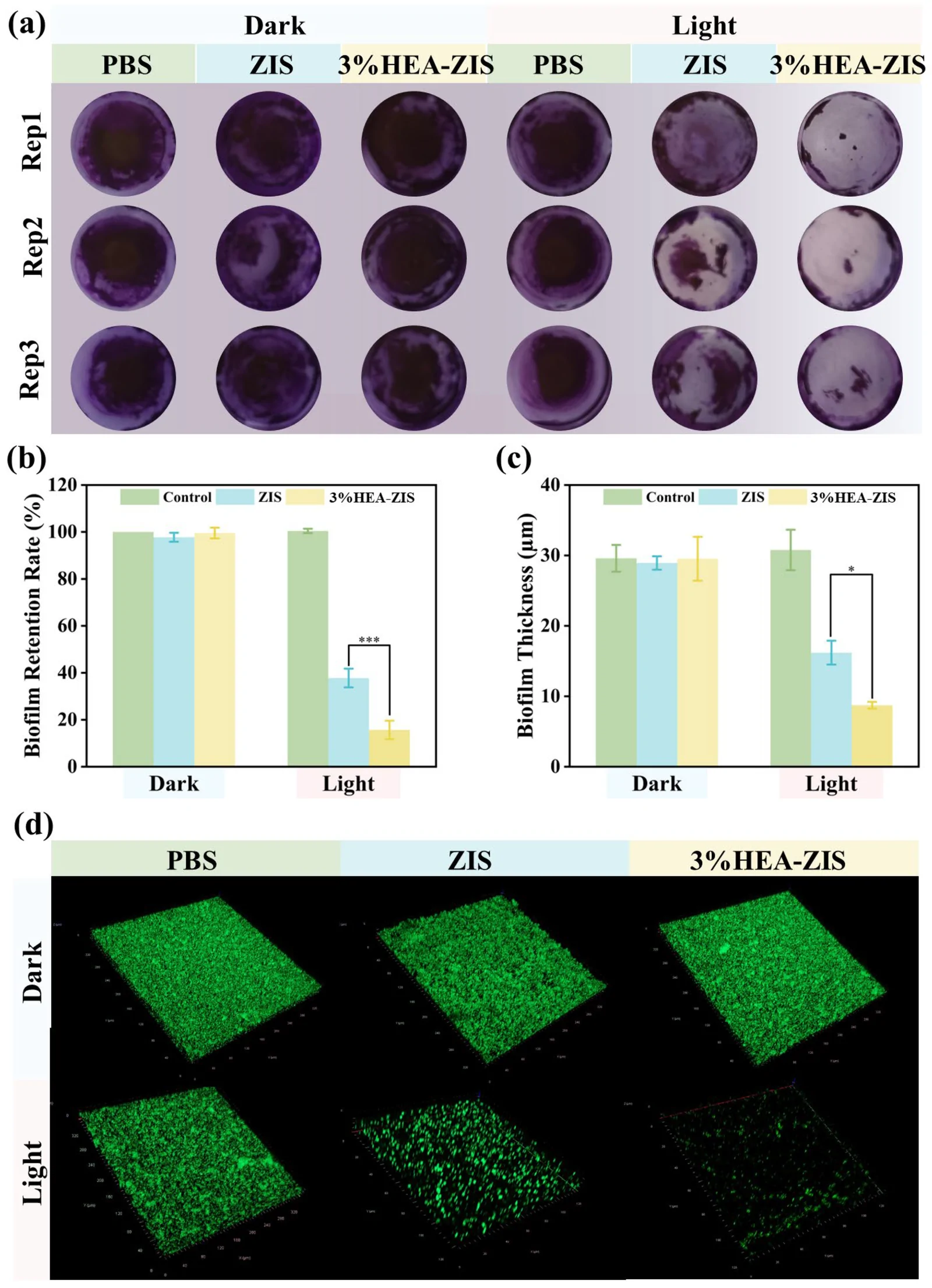
Evaluation of anti-biofilm activity. (**a**) Representative images of crystal violet-stained biofilms under different treatments. (**b**) Quantitative analysis of residual biofilm biomass. *n* = 2, *** *p* < 0.001. (**c**) Statistical analysis of the average biofilm thickness derived from CLSM images. *n* = 2, * *p* < 0.05. (**d**) Representative 3D reconstructed CLSM images of MRSA biofilms stained with SYTO9.

**Figure 5:**
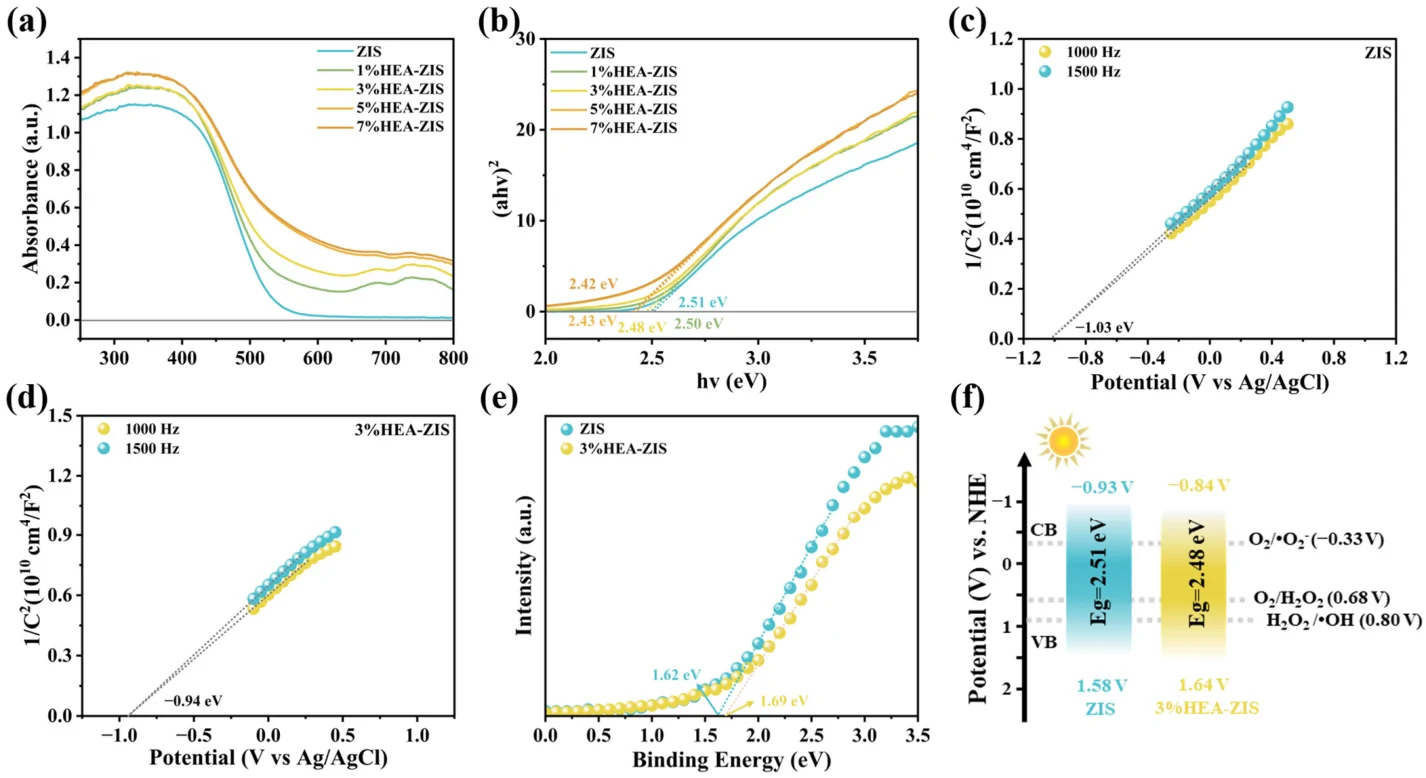
(**a**) UV-vis DRS spectra of ZIS and HEA-ZIS composites with different HEA loadings. (**b**) Corresponding Tauc plots for band gap estimation. (**c**,**d**) Mott–Schottky plots of ZIS (**c**) and 3%HEA-ZIS (**d**) measured at different frequencies. (**e**) XPS valence band spectra of ZIS and 3%HEA-ZIS. (**f**) Schematic band structure diagram of ZIS and 3%HEA-ZIS.

**Figure 6:**
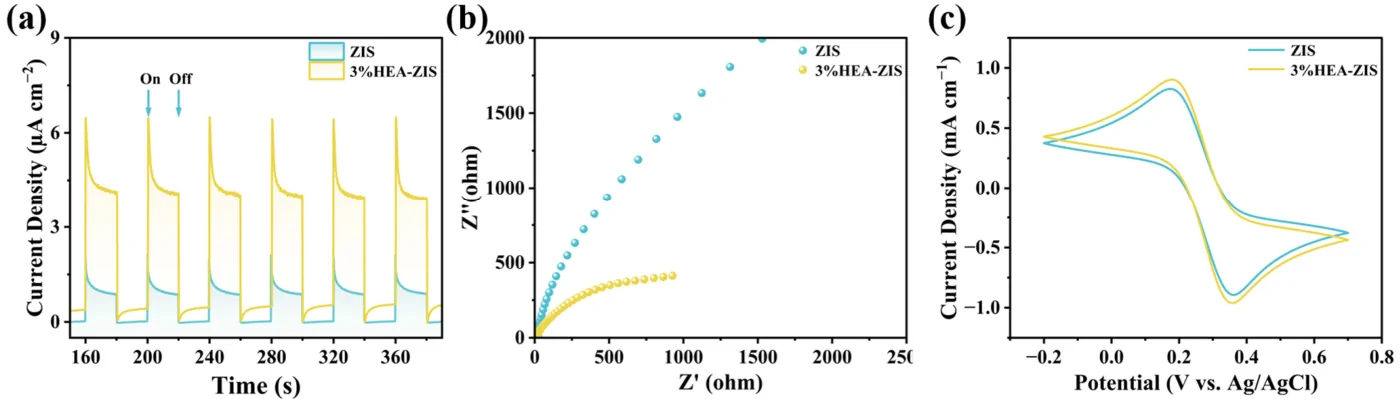
Photoelectrochemical properties of the samples. (**a**) Transient photocurrent responses of ZIS and 3%HEA-ZIS under intermittent light irradiation. (**b**) Nyquist plots from electrochemical impedance spectroscopy. (**c**) Cyclic voltammetry curves of ZIS and 3%HEA-ZIS.

**Figure 7:**
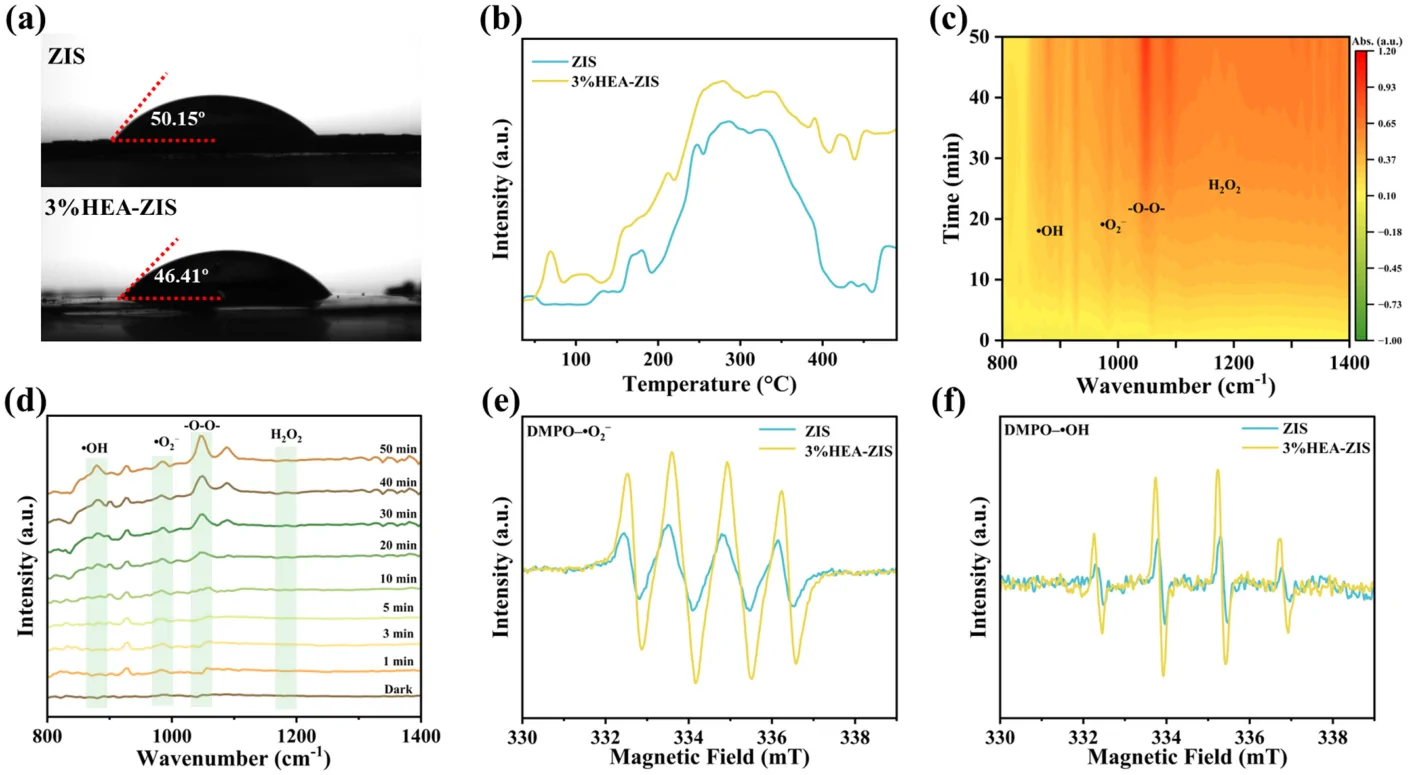
Surface physicochemical properties and ROS characterization. (**a**) Water contact angles of ZIS and 3%HEA-ZIS. (**b**) O_2_-TPD profiles of ZIS and 3%HEA-ZIS. (**c**,**d**) In situ DRIFTS spectra of 3%HEA-ZIS at varying light irradiation intervals. (**e**,**f**) EPR spectra of (**e**) •O_2_^−^ and (**f**) •OH trapped by DMPO over ZIS and 3%HEA-ZIS after 5 min of simulated sunlight irradiation.

## Data Availability

All data needed to support the conclusions in the paper are presented in the manuscript and Supplementary Materials. Further inquiries can be directed to the corresponding authors.

## Supplementary Materials

The following supporting information can be downloaded at https://www.mdpi.com/article/10.3390/ijms27209073/s1.

## Abbreviations

The following abbreviations are used in this manuscript:

ARB	Waterborne antibiotic-resistant bacteria
ROS	Reactive oxygen species
ZnIn_2_S_4_	Zinc indium sulfide
•O_2_^−^	Superoxide radicals
•OH	Hydroxyl radicals
^1^O_2_	Singlet oxygen
HEAs	High-entropy alloys
MRSA	Methicillin-resistant *Staphylococcus aureus*
CFU	Colony-Forming unit
MIC	Minimum inhibitory concentration
MBC	Minimum bactericidal concentration
TSA	Tryptic soy agar
CLSM	Confocal laser scanning microscopy
DRS	UV-vis diffuse reflectance spectroscopy
VB	Valence band
CB	Conduction band
