# Supplementary material for: High-Entropy Alloy-Engineered ZnIn2S4 Photocatalyst for Efficient In Vitro Eradication of MRSA and Inhibition of Biofilm Formation via ROS-Mediated Mechanisms
Source: Int J Mol Sci. 2026 Oct 13;27(20):9073. doi: 10.3390/ijms27209073 (PMC13705492; doi:10.3390/ijms27209073)
Supplement: Supplementary file 1 [file ijms-27-09073-s001.zip › ijms-4318450-supplementary.pdf]

International Journal of *Molecular Sciences*

MDPI

Article

High-Entropy Alloy-Engineered $ZnIn_2S_4$ Photocatalyst for Efficient In Vitro Eradication of MRSA and Inhibition of Biofilm Formation via ROS-Mediated Mechanisms

Xuehua Chen [1], Tai-Cheng Zhou [2], Changle Wen [1], Ying Zhang [3], Hao Wang [4,*] and Jia Wei [1,*]

[1] School of Medicine, Yunnan University, Kunming 650091, China; chenxh1117_edu@163.com (X.H.C.); wenchangle2000@163.com (C.L.W.); weijia@ynu.edu.cn (J.W.)
[2] Central Laboratory, The Affiliated Hospital of Yunnan University, Kunming 650021, China; zhoutc@ynshhyy.com
[3] Key Laboratory of Bioinorganic and Synthetic Chemistry of the Ministry of Education, Fine Chemical Industry Research Institute, School of Chemistry, Sun Yat-Sen University, Guangzhou 510275, China; zhangy656@mail2.sysu.edu.cn
[4] Faculty of Chemical Engineering, Kunming University of Science and Technology, Kunming 650500, China
* Correspondence: haowang668@kust.edu.cn (H.W.); weijia@ynu.edu.cn (J.W.)

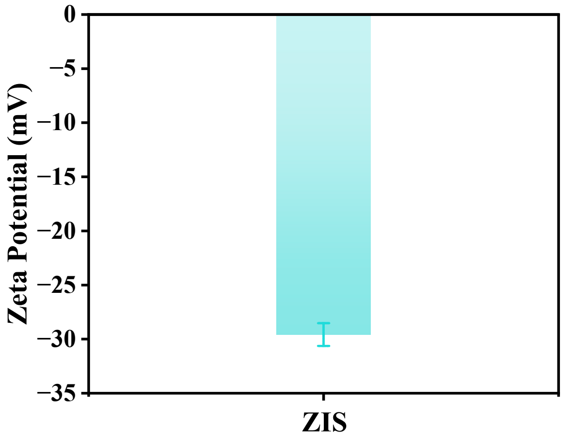

Figure S1. Zeta potential of ZIS.

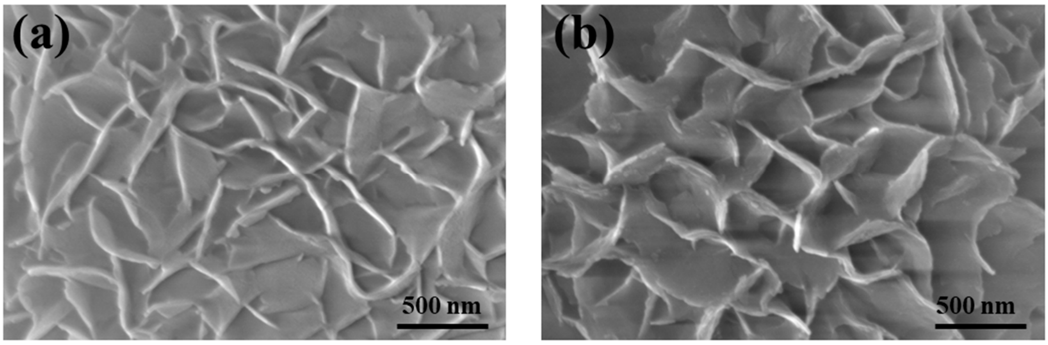

Figure S2. FESEM images of (a) pristine ZIS and (b) 3%HEA-ZIS.

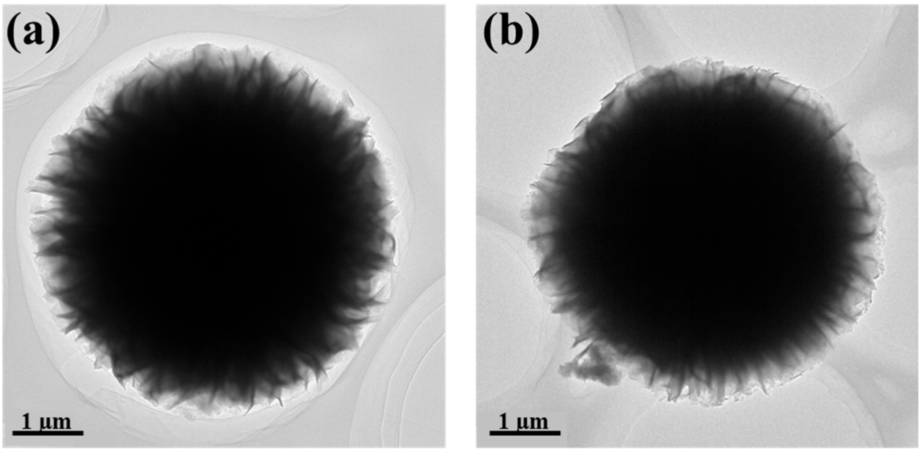

Figure S3. TEM images of (a) pristine ZIS and (b) 3% HEA-ZIS.

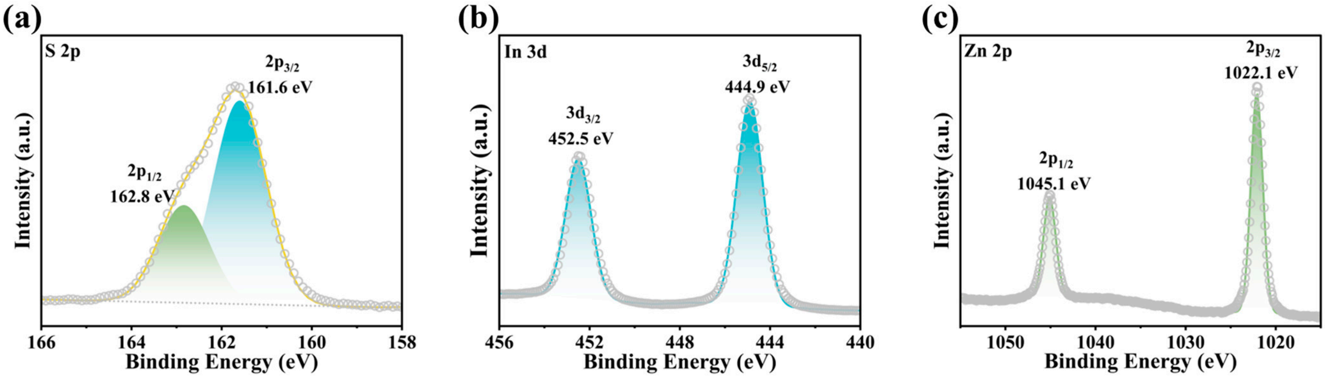

Figure S4. High-resolution XPS spectra of 3%HEA-ZIS: (a) S 2p, (b) In 3d, (c) Zn 2p.

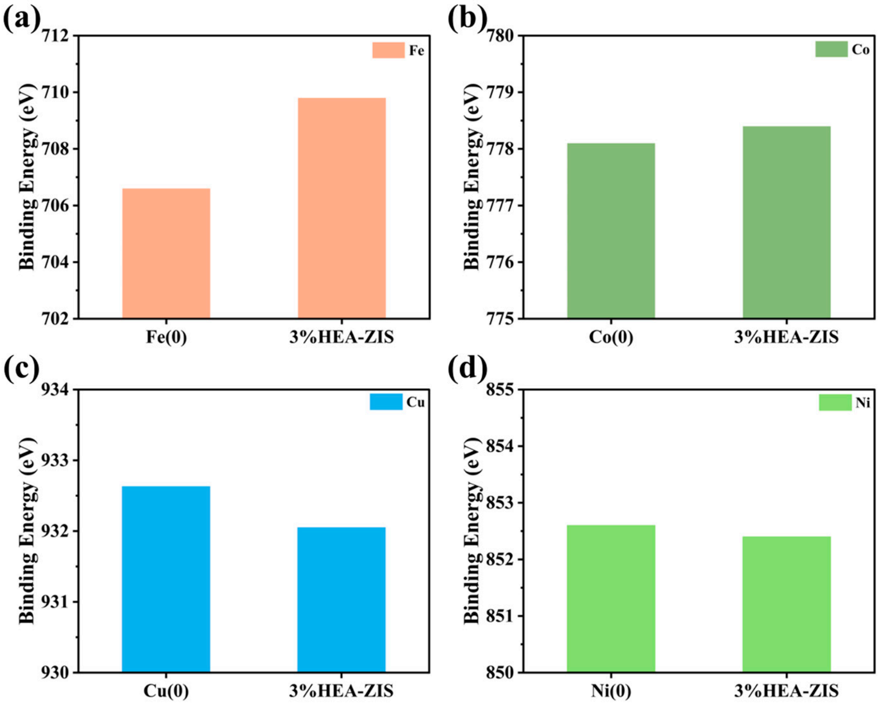

Figure S5. A comparison of the binding energies of (a) Fe, (b) Co, (c) Cu, and (d) Ni in 3% HEA-ZIS with the standard values of their respective metallic states.

Table S1. Elemental compositions of HEA–ZIS at different HEA loadings as determined by ICP-OES.

	1% HEA-ZIS	3% HEA-ZIS	5% HEA-ZIS	7% HEA-ZIS
Fe	0.667	3.507	4.529	7.949
Cu	1.06	4.136	6.539	10.655
Ni	1.209	3.664	5.414	9.051
Zn	14.1692	14.3301	13.964	13.3872
Co	1.19	3.35	4.778	7.732

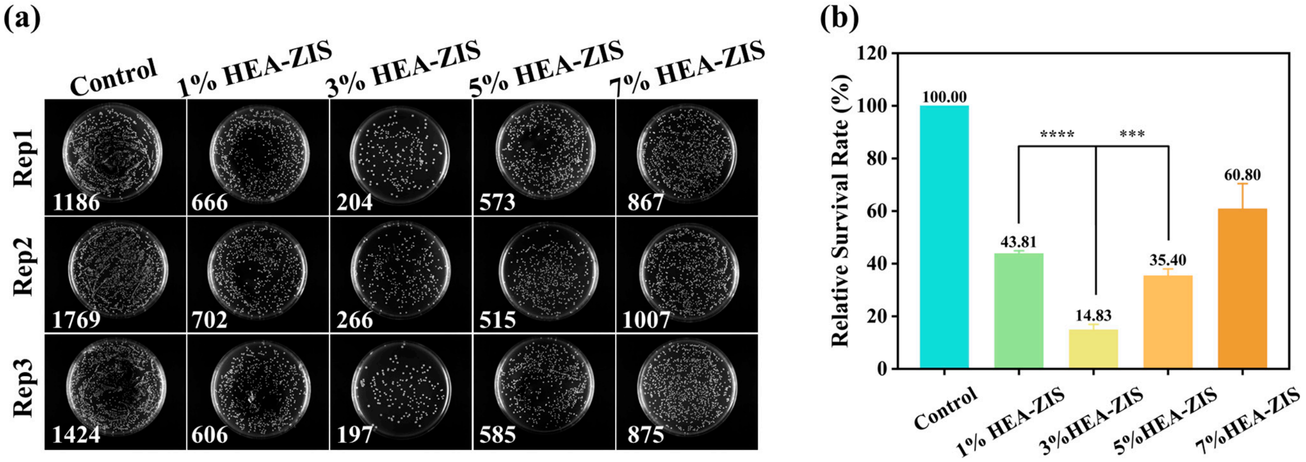

Figure S6. Antibacterial efficacy of HEA–ZIS with different HEA contents. (a) Photographs showing the colony formation of MRSA on TSA plates after indicated treatments. (b) Corresponding survival rates calculated from colony counting assays. *n*=3, ***p<0.001, ****p<0.0001.

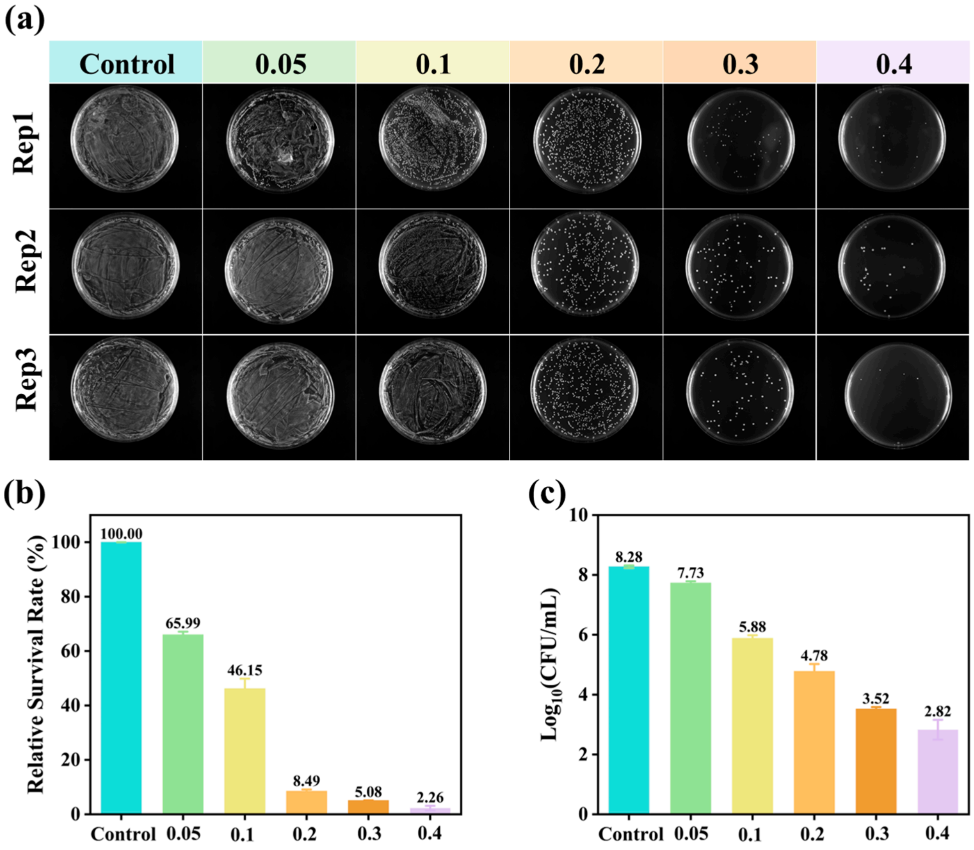

Figure S7. Dose-dependent antibacterial activity of 3% HEA-ZIS against MRSA. (a) Photographs of MRSA colonies on TSA plates after treatment with varying dosages of the catalyst. (b) Inactivation efficiency as evaluated by optical density at 600 nm (OD_{600}) (n = 3). (c) Bacterial survival levels quantified as log_{10}(CFU/mL) (n = 3).

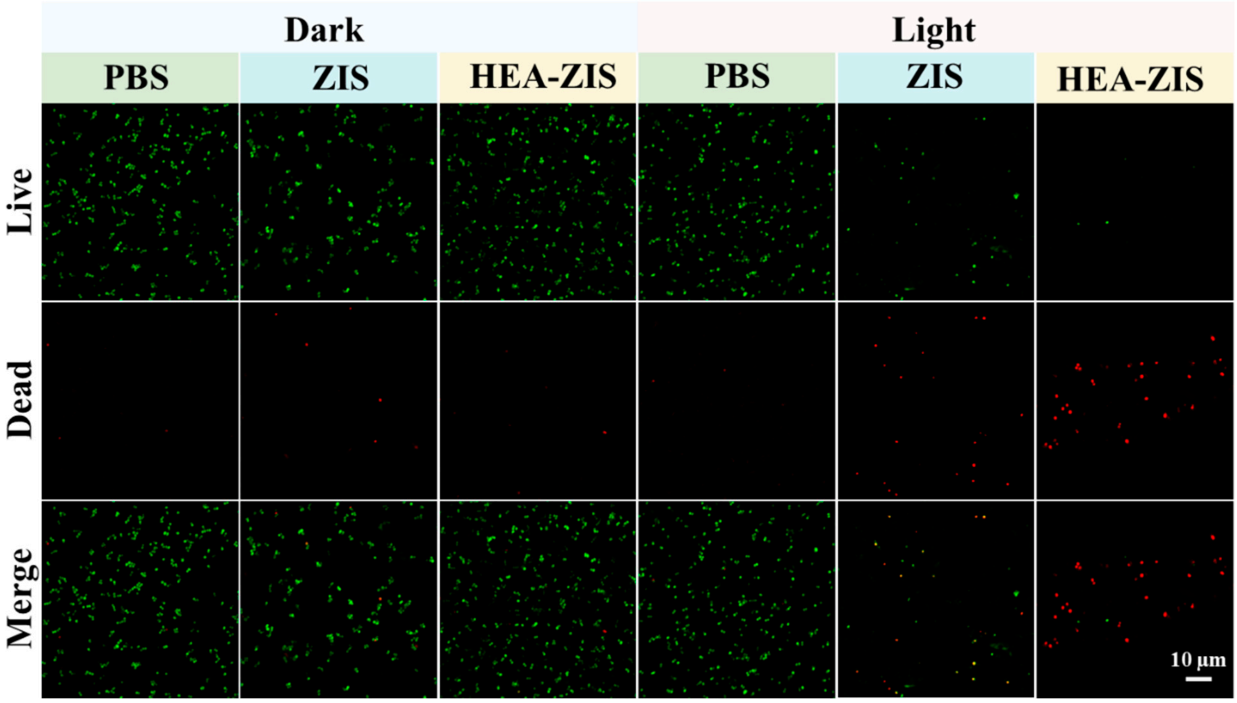

Figure S8. CLSM images of MRSA after different treatments stained with SYTO 9/PI live/dead assay (live, green; dead, red).

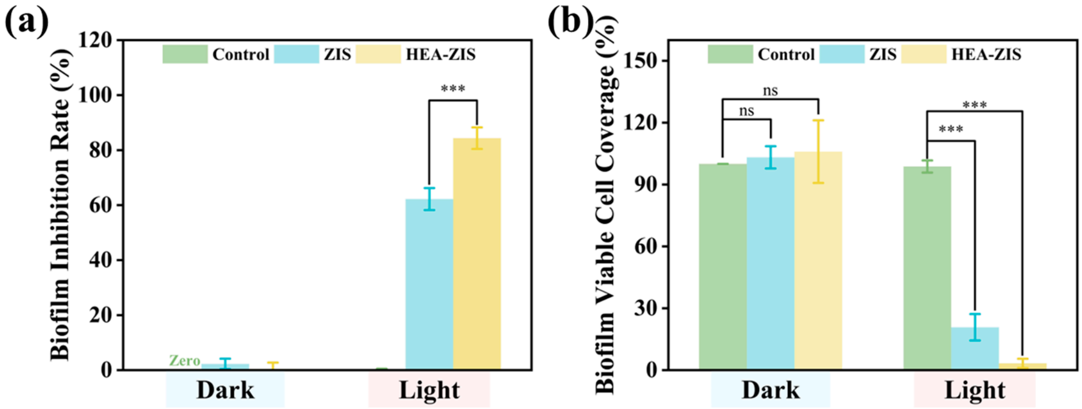

Figure S9. (a) Percentage of biofilm inhibition evaluated by the crystal violet method. n=2, ***p<0.001. (b) Spatial distribution of viable cells based on SYTO9-labeled bacterial membrane coverage. n=2, ns: indicates a non-significant difference, ***p<0.001.

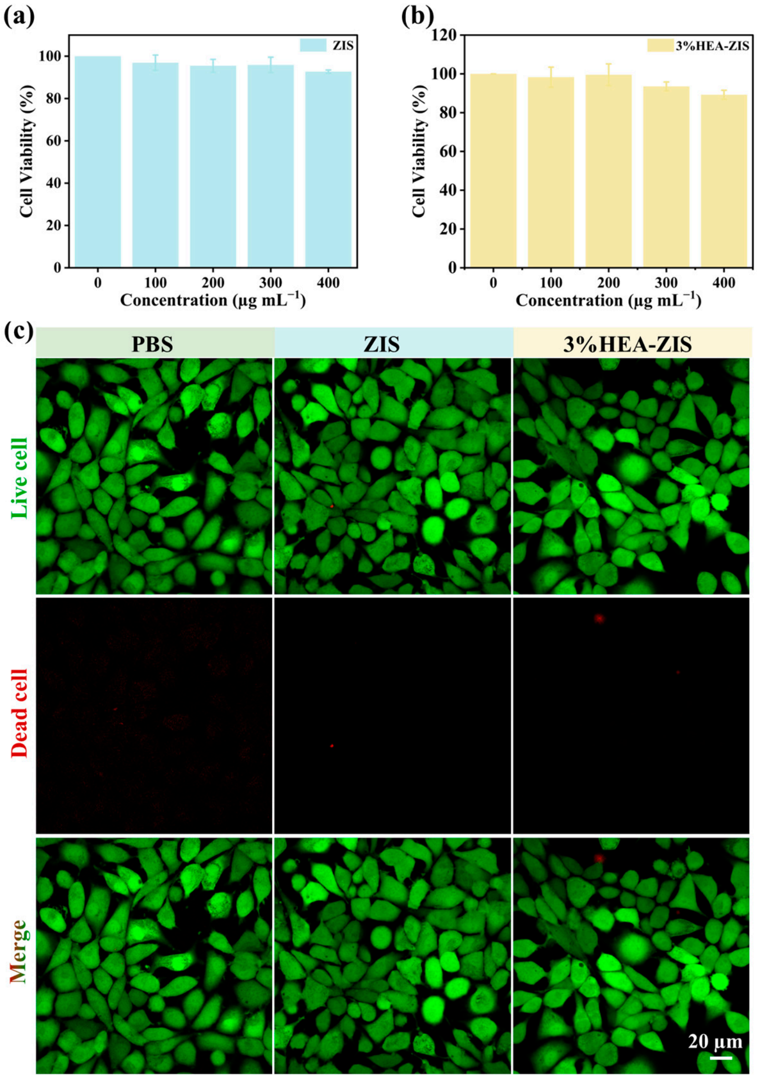

Figure S10. In vitro biocompatibility of ZIS and 3% HEA-ZIS. (a, b) Cell viability of L929 cells after 24 h incubation with ZIS (a) and 3% HEA-ZIS (b); *n*=3. (c) Live/dead staining images of L929 cells after 24 h incubation with ZIS and 3% HEA-ZIS. Green indicates live cells, and red indicates dead cells.

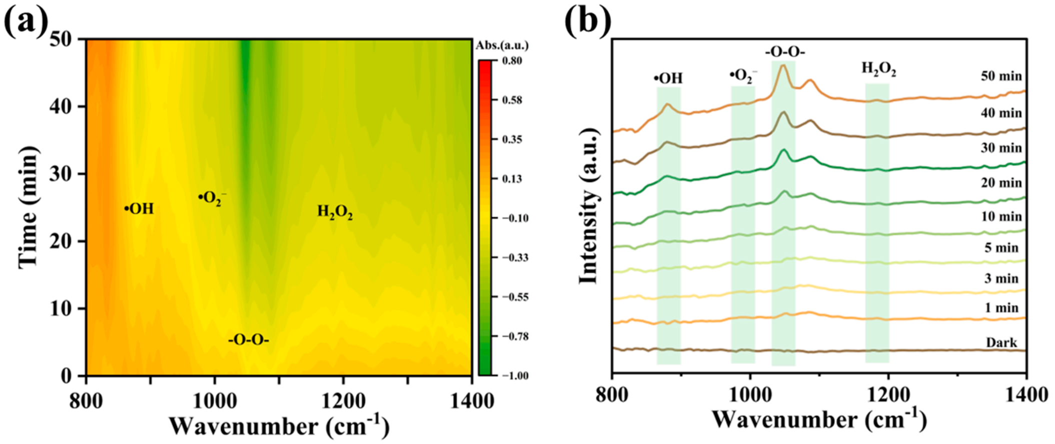

Figure S11. (a, b) In situ DRIFTS spectra of ZIS collected at varying light irradiation intervals.
